# Pauses in Striatal Cholinergic Interneurons: What is Revealed by Their Common Themes and Variations?

**DOI:** 10.3389/fnsys.2017.00080

**Published:** 2017-10-30

**Authors:** Yan-Feng Zhang, Stephanie J. Cragg

**Affiliations:** ^1^Department of Physiology, Anatomy and Genetics, University of Oxford, Oxford, United Kingdom; ^2^Oxford Parkinson’s Disease Centre, University of Oxford, Oxford, United Kingdom

**Keywords:** tonically active neuron, cholinergic interneuron, pause response, striatum, dopamine, cortex, thalamus

## Abstract

Striatal cholinergic interneurons, the so-called tonically active neurons (TANs), pause their firing in response to sensory cues and rewards during classical conditioning and instrumental tasks. The respective pause responses observed can demonstrate many commonalities, such as constant latency and duration, synchronous occurrence in a population of cells, and coincidence with phasic activities of midbrain dopamine neurons (DANs) that signal reward predictions and errors. Pauses can however also show divergent properties. Pause latencies and durations can differ in a given TAN between appetitive vs. aversive outcomes in classical conditioning, initial excitation can be present or absent, and a second pause can variably follow a rebound. Despite more than 20 years of study, the functions of these pause responses are still elusive. Our understanding of pause function is hindered by an incomplete understanding of how pauses are generated. In this mini-review article, we compare pause types, as well as current key hypotheses for inputs underlying pauses that include dopamine-induced inhibition through D_2_-receptors, a GABA input from ventral tegmental area, and a prolonged afterhyperpolarization induced by excitatory input from the cortex or from the thalamus. We review how each of these mechanisms alone explains some but not all aspects of pause responses. These mechanisms might need to operate in specific but variable sets of sequences to generate a full range of pause responses. Alternatively, these mechanisms might operate in conjunction with an underlying control mechanism within cholinergic interneurons which could potentially provide a framework to generate the common themes and variations seen amongst pause responses.

## Introduction

The so-called tonically active neurons (TANs) in the striatum of the basal ganglia in behaving monkeys are thought to be the cholinergic interneurons (ChIs; Aosaki et al., 2010). Although only 1%–2% striatal neurons are ChIs, these neurons provide a major source of acetylcholine to the striatum and are involved in acutely regulating striatal output as well as striatal learning (Aosaki et al., 1994b, 1995, 2010; Ravel et al., 2003; Witten et al., 2010; Aoki et al., 2015). In the 1980s and early 1990s, TANs were considered “poorly modulated cells”, because they fire action potentials regularly during movements (Kimura et al., 1984; Apicella et al., 1991). In parallel, they also demonstrate strong pacemaker activity *ex vivo* in slices that is only weakly modulated (Bennett et al., 2000). It was not until 1994 that Aosaki et al. (1994b) found that tonic firing neurons form a dynamic “pause response”, a transient reduction in firing rate, following a sensory cue which indicates a reward. We now know that this dynamic activity can be timelocked to phasic changes in DA neuron activity, reinforcing the growing literature that suggests ACh and DA systems interact in processing and learning about rewards (Morris et al., 2004; Cragg, 2006; Joshua et al., 2008; Goldberg and Reynolds, 2011). However, much remains to be understood about the pause in TAN activity and there are many open questions about its features, functions and mechanisms of formation. This mini-review article will discuss the common and divergent characteristics of pause responses, and whether known mechanisms can account for these features.

## What Is A Pause Response in TANs?

**Figure 1:**
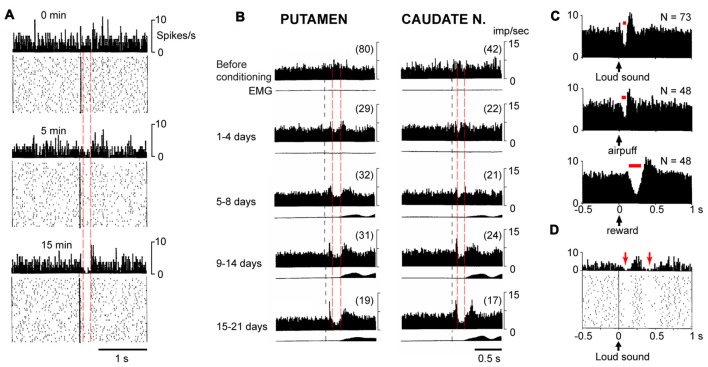
Different pause responses in tonically active neurons (TANs) *in vivo* show divergent and constant properties.** (A)** Development of a pause response in a typical TAN during a new conditioning at times 0, 5 and 15 min. The pause develops in amplitude but not in latency or duration (*dashed red lines*). Each panel represents 5 min of recording, *upper*, peri-stimulus time histogram (PSTH), *lower*, rasters of individual sweeps. Adapted with permission from Aosaki et al. (1994b). **(B)** Development of a pause response in a population of TANs *in vivo* (*n* = 17–80) during conditioning over days. Pauses in the peri-stimulus time histogram (PSTH) develop in amplitude but not latency and duration (*dashed red lines*), in monkey putamen (*left*) and caudate nucleus (*right*). *Lower trace* in each panel shows the movement (EMG) which occurs later than the pause. Adapted, with permission, from Aosaki et al. (1994b). **(C)** Pause responses in a population of TANs in the same animal have different latency and duration (*red bar*) when a loud sound, air puff, or reward were applied. Adapted with permission from Ravel et al. (2003). **(D)** A TAN can show two pauses (*red arrows*) after a stimulus (loud sound, *black* *arrow*) is applied, PSTH (*upper*) and raster data (*bottom*). Adapted with permission from Ravel et al. (2003).

Pause response in TANs were first found in monkeys during classical conditioning experiments in which an audible click indicated the subsequent delivery of a reward (Aosaki et al., 1994a). When the firing activities of TANs on each trial were aligned to the auditory cue, the peri-stimulus time histogram (PSTH) showed that the TANs respond to the cue with an initial excitation (or a burst) about 60 ms later, followed by a pause at about 90 ms which lasted for about 200 ms, followed by an excitation which was named rebound activity (Figures 1A,B). Similar triphasic pause responses in TANs have now been well-documented by other labs and observed in other behavioral paradigms (Matsumoto et al., 2001; Shimo and Hikosaka, 2001; Ravel et al., 2003; Morris et al., 2004; Joshua et al., 2008; Apicella et al., 2009, 2011). The pause responses observed across various paradigms can share many common characteristics but they can also show divergent characteristics in different tasks. To note, although the term “pause response” was coined on its first observation (Aosaki et al., 1994b), the pause phase is not necessarily an absolute silencing of the firing activities at the population level, but can be a transient reduction in firing rate (Figures 1A,B). Hence the variable features of the pause can include a change in amplitude, likely reflecting a change in the number of local ChIs that show a silencing. An understanding of the various commonalities and differences seen in pause responses should help to shed light on their functions and causes.

### Common Characteristics in Pause Responses

#### Pause Responses Are Synchronized and Become Amplified Across a Population of Neurons

TANs can acquire a pause response during learning at the single cell level as well as on a population level. Aosaki et al. (1994a) reported that about 17% of TANs responded to a click-cue with a pause without any training, but that with ongoing training, up to 73% of TANs across the striatum responded. The development of pause responses observed in population activity is promoted by dopamine. Unilateral lesioning of nigrostriatal dopamine pathways using 1-methyl-4-phenyl-1,2,3,6-tetrahydropyridine (MPTP), which causes irreversible dopamine deficits, reduced the number of neurons showing pauses back to baseline level (18.7%), whilst apomorphine, a non-selective dopamine receptor agonist reversed these effects (Aosaki et al., 1994a).

During learning, the amplitude of the pause response becomes more profound at the single cell level (Figure 1A) as well as at the population level (Figure 1B). However, as the pause develops, it changes in amplitude without changes in latency or duration for each of the pause response phases (Figures 1A,B). This characteristic means that the TANs that pause, do so in synchrony, regardless of the progress of learning. The available evidence corroborate that the latency and duration of pause responses remains the same during learning (Aosaki et al., 1994b), although studies have not to our knowledge systematically excluded the alternative possibility that pause responses are asynchronous across neurons and become more synchronized through learning.

Synchrony of each component of the triphasic pause response across the TAN population is one of the unique characteristics of pause responses. That means TANs are not only pausing in synchrony but also have initial excitation and rebound of similar latency and duration (Aosaki et al., 1994b, 1995; Apicella et al., 1997; Ravel et al., 2003). Such synchrony is particularly surprising because the TANs are so sparsely distributed. This suggests that pause responses in TANs are driven by a signal that is widely available or broadcasted throughout the striatum.

There are likely to be implications of synchronized changes in ChI firing for acetycholine function. The triphasic pause response is thought to provide a corresponding high-low-high pattern of availabilty of acetycholine. Fast catabolism of acteylcholine by acetylcholinesterase (Quinn, 1987) should be sufficient to clear acetylcholine within the 200 ms of the pause phase. This fluctuation of acetylcholine level in the striatum and its timing relative to the salient sensory cue and reward is believed to play an important role in striatal signaling and learning and by providing a time window for a change to postsynaptic integration and to dopamine release (Morris et al., 2004; Cragg, 2006; Aosaki et al., 2010; Goldberg and Reynolds, 2011).

#### Pause Responses in Relation to Dopamine Neuron Activity

By simultaneously recording from TANs in the striatum and dopamine neurons (DANs) in the SNc during a classcial conditioning task, Morris et al. (2004) demonstrated that the pause phase of TANs coincides with phasic firing of DANs, in response to conditioned cues as well as to reward prediction errors. However, when TANs respond with a pause, i.e., a negative change in firing rate, DANs by contrast reflect the mismatch between expectation and outcome, i.e., the prediction error, through both positive and negative changes in phasic firing rate (Schultz et al., 1997; Morris et al., 2004). Joshua et al. (2008) further showed that DANs responded more strongly to appetitive than neutral or aversive cues, whereas the TAN responded similarly to all cue types. Therefore, although the latencies of responses of TANs and DANs are coincident, these two types of neurons do not represent external information in the same way indicating that the dual representation of reward prediction-related information by TANs and DANs is not redundant.

### Not All Pause Responses Are the Same

The pause responses seen during different events can share characteristics outlined above, but they also have variations. A “typical” pause response is seen when an animal receives a reward after a sensory cue. However, when the appetitve outcome is replaced by an aversive outcome, the duration of pause response is shorter (161 ms vs. 78 ms respectively; Ravel et al., 2003; Figure 1C). In addition, TANs (and also DANs) responded with shorter latency to aversive stimuli than to appetitive (food) outcome (Joshua et al., 2008). Importantly, these different pause timings can be observed in the same TANs (Ravel et al., 2003), which indicates that rather than being regulated by intrinsic properties alone, pause duration is likely also to be governed by inputs.

Pause responses occur in some but not all of TANs. As described above, without training, sensory stimuli and free reward can cause a pause response in a small proportion of TANs (10%–20%; Aosaki et al., 1994a) but when the animals are well trained, more TANs (80%) respond. But TANs that respond to one task may not be responsive to another. For example, 65% of TANs responded to one or two aspects of classical conditioning, free reward, or instrumental tasks, but only 24% of neurons responded to all three events (Apicella et al., 1997). Furthermore, the TANs that respond with a pause in a given task can be distributed in a scattered fashion throughout the striatum. Two neighboring TANs may not respond in the same way to the same stimuli (Aosaki et al., 1994b; Ravel et al., 1999; Apicella et al., 2009). Therefore, the driving force of the pause response is likely to be a widely distributed or synchronized input but with a differential outcome.

Not all pause responses have three phases. The initial excitation that can precede a pause occurs in only about the half of pause responses (Aosaki et al., 1994b). On the other hand, a post-excitation “rebound” is usually observed (Aosaki et al., 2010). Interestingly, a second pause and rebound have also been recorded (Ravel et al., 2003, 2006; Apicella et al., 2011; Doig et al., 2014; Figure 1D).

A pause does not always mean an absolute silencing of TANs. In many cases, the pause phase is a period when TANs fire action potentials at a slower rate (than baseline) instead of falling fully silent (Aosaki et al., 1995; Ravel et al., 2003). Action potentials can occur within the pause phase (Figure 1A). This observation suggests that the mechanism(s) underlying the pause response can depress the firing rate of TANs for a period longer than one cycle of action potential and its afterhyperpolarization (AHP).

Thus, the variations in pause responses seen to date have some common features and some key differences. They usually share the characteristics of synchrony, constant latency and duration during learning of a given response, and coincidence with phasic activities of midbrain DANs. They show divergence in latency and duration when the outcome of classical conditioning is changed, in the responses of different neurons to different stimulus types and in the presence of the initial excitation. Given these observations, can we decipher what information is present in the pause response?

## What Information Do Pause Responses Encode?

To investigate the information carried by pause responses, TANs have been recorded during a range of experimental tasks. By correlating the firing pattern of the TANs and the parameters of the experiments, it has been shown that pauses occur in response to a large range of events that include sensory cues, prediction errors and primary rewards in classical conditioning, instrumental task and free reward situation (Apicella et al., 1997, 2009, 2011; Yamada et al., 2004; Nougaret and Ravel, 2015) as well as as aversive stimuli (Ravel et al., 1999, 2003). Pause amplitude has been shown to present some spatial and temporal information about the cue and reward (Sardo et al., 2000; Ravel et al., 2001, 2006; Shimo and Hikosaka, 2001; Lee et al., 2006). In addition, the rebound phase of the pause response has a variable amplitude which is correlated to reward probability (Apicella et al., 2011). These findings suggest the different components of the “pause response” might play distinct functional roles.

However, without knowing what is driving pause responses in TANs, it is hard to understand what information they carry. Several mechanisms have been proposed to underlie pause responses, with each focussed on particular aspects of the pause response. Various input regions have been suggested to be possible sources of the pause response, including the midbrain, the cortex, the thalamus, and striatum itself. We review these proposed contributing sources of the pause response here. It should however be noted that while the pause responses in TANs have mainly been studied in monkeys, the underlying candidate mechanisms have predominantly been explored in rodents.

### Midbrain Dopamine Input to ChIs

Dopamine has been considered a major potential driving force of the pause response because dopamine is necessary for pause response to develop into more TANs following training (Aosaki et al., 1994a). Reynolds et al. (2004) demonstrated *in vivo* that pauses in the firing of striatal ChIs can be induced by electrical stimulation of the SNc DANs in rats. There is evidence that expression of acquired pause responses *in vivo* involves D_2_-type and D_1_-type receptors (Watanabe and Kimura, 1998). *Ex vivo* in slices, minimizing D_2_ currents in particular, either pharmacologically (Ding et al., 2010), by a prior 6-OHDA lesion (Sanchez et al., 2011) or genetically (Kharkwal et al., 2016), can diminish the duration or amplitude of the pause induced by electrical stimulation. Besides corroborating a dopamine-dependence of pause expression, these findings also highlight that under parkinsonian conditions, ChI excitability is likely to be higher and with reduced pausing.

**Figure 2:**
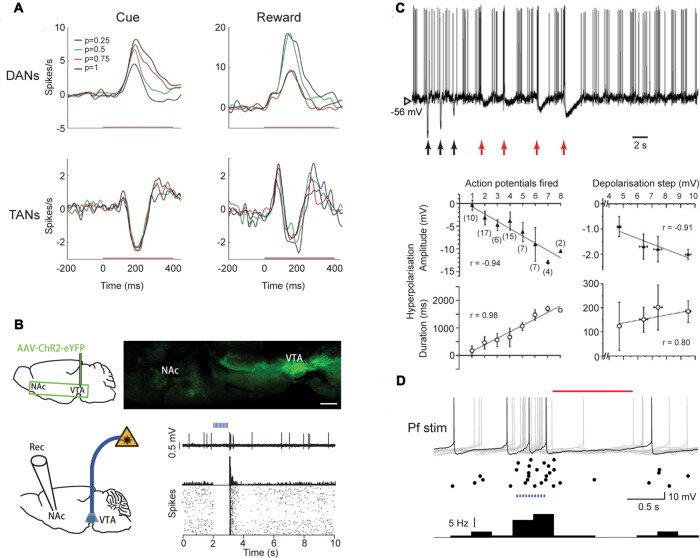
Regulation of pause responses by inputs. **(A)** Dopamine neurons (DANs) and TANs respond to the cue and reward at similar latencies and duration but in opposite directions. Reward probability (*color coded*) modifies firing rate in DANs but not in TANs. Adapted from Morris et al. (2004). **(B)** Schematic diagram (*upper left*) and sagittal brain section (*upper right*) showing GABA neurons in VTA (GAD-Cre1 mouse) that project eYFP1-expressing axons to NAc. Activation of GABA neurons in VTA via an optic fiber (4-ms pulses, 20 Hz for 1 s) inhibits a ChI in NAc (PSTH, 5 ms bins and raster plots *bottom right*). Adapted with permission from Brown et al. (2012). **(C)** *Upper*, Depolarization (*red arrows*, 0.2, 0.4, 0.6, 0.8 nA), but not hyperpolarization (*black arrows*, −0.6, −0.4, −0.2 nA), of a ChI in the striatum *in vivo* induces a prolonged AHP. *Lower*, the amplitude and duration of the AHP is proportional to number of evoked action potentials number and depolarization step size. Adapted from Reynolds et al. (2004). **(D)** A pause in firing in ChIs (*red line*) in slices is induced following optogenetic activation *(blue dots)* of Pf afferents (400 ms train, 25 Hz). *Upper*, example current clamp traces superimposed (*gray*), with 1 typical trace highlighted (*black*), and *middle*, raster plot and *lower*, histogram (bin size 0.25 s, bottom). Adapted with permission from Kosillo et al. (2016).

However, a D_2_ receptor-induced pause does not obviously explain key aspects of pauses. For example, if the pause phase results from acute D_2_ receptor activation during concurrent activity in DANs, the amplitude of the pause should correlate to the activities of DANs. On the contrary, the pause response does not co-vary with the frequency of DAN activity (Morris et al., 2004; Joshua et al., 2008; Figure 2A). The TANs can also pause following a sensory cue when the reward probability is zero and DANs are not activated (Morris et al., 2004). Therefore, the TANs can pause regardless of the coincident firing pattern of the DANs. Notably, dopamine depletion with MPTP does not eliminate the pause response in all TANs but rather, prevents the development of the pause response in a larger population of TANs (Aosaki et al., 1994a). In addition, the extremely branched and overlapping arbors of extensive dopamine axons (Matsuda et al., 2009) along with a volume transmission mode of action by dopamine (Cragg and Rice, 2004), make it hard to understand why dopamine would induce pauses in only some TANs but not other neighboring ones. Yet further, the depression of ChI firing rate induced by D_2_ current does not explain how the pause might be preceded by an initial excitation. Therefore, dopamine might play a more important role in the development of the pause response rather than in its acute driving. To support this hypothesis, dopamine has been shown to promote long-term potentiation in excitatory inputs to ChIs (Suzuki et al., 2001; Reynolds et al., 2004).

Co-transmission of glutamate from DANs (Stuber et al., 2010; Tecuapetla et al., 2010) has recently been show to excite ChIs (Chuhma et al., 2014; Wieland et al., 2014) and could potentially contribute to an initial excitation phase prior to a pause response. However, the timing of phasic activities in DANs and TANs does not appear to support such a mechanism, since phasic activity in DANs coincides with the later TAN pause phase but not the initial excitation (Morris et al., 2004; Joshua et al., 2008). In addition, the co-release of glutamate is reported to be particularly evident in ventral striatum (Tecuapetla et al., 2010) which can not explain the initial excitation of the TANs in the dorsolateral striatum.

Co-transmission of GABA from DANs has also been found, in both ventral and dorsal striatum (Tritsch et al., 2012, 2014). However, direct optogenetic activation of GABA co-release from dopamine terminals does not result in a short latency inhibition of ChIs (Straub et al., 2014).

### Midbrain GABA Input to ChIs

A population of GABA neurons in the VTA has been identified that target striatal ChIs. Activating this group of GABA neurons using optogenetic methods can induce a pause and a rebound in the ChIs in the ventral striatum (Brown et al., 2012; Figure 2B). However, a GABAergic mechanism has not been described for dorsal striatum. In addition, GABA neurons in VTA have been shown to exhibit sustained activation from the sensory cue to the reward, which does not match the time course of the pause response in TANs (Cohen et al., 2012). Therefore, the contribution of these GABAergic neurons *in vivo* during behavior might not be critical to pause responses.

### Cortical Input to ChIs

Corticostriatal inputs form synaptic contact with ChIs at their distal dendrites (Thomas et al., 2000; Doig et al., 2014). Electrical stimulation *in vivo* of either contralateral Reynolds et al., 2004 or ipsilateral cortex (Doig et al., 2014) induces an initial excitation in rat ChIs, and a subsequent reduction in membrane excitation that underlies a period of reduced firing. Activating ChIs by stimulating axons from cortical neurons with an optogenetic method can also induce initial excitation followed by a subsequent pause in slices (Kosillo et al., 2016). Cortical input is therefore a good candidate for a source signal of pause responses because it is able to induce the initial excitation, a pause phase and sometime a rebound in TANs.

*In vivo and ex vivo* slice experiments have suggested that the pause induced by the cortical stimulation is determined by the AHP (Reynolds et al., 2004; Oswald et al., 2009, 2015). However, the AHP duration is variable and is proportional to the stimulation intensity/initial excitation *in vitro* and *in vivo* (Reynolds et al., 2004; Oswald et al., 2009; Figure 2C). By contrast, learning *in vivo* does not induce a longer pause (Figure 1). In addition, the stimulation strength does not need to be above a threshold for generating spiking in order to be able to induce a lower firing rate (Reynolds et al., 2004). Furthermore, as suggested by the same group, the particular AHP induced by initial excitation is caused by an I_h_ current that is active when membrane potential is higher than its reversal potential (−40 or −20 mV; Oswald et al., 2009). Because this driving force to hyperpolarize ChIs will disappear when the membrane potential falls below the reversal potential of I_h_, it would be expected to influence only one interspike interval. Therefore, AHP induced by an initial excitation seems unable to account for the pause being a period of reduced firing rate, rather than a total suppression of firing (Figure 1D). If not through a prolonged AHP, through what mechanism could cellular excitability account for the pause?

### Thalamic Input to ChIs

Thalamic inputs are also key candidates for contributing to pause generation, in several respects. Compared to corticostriatal inputs, thalamostriatal inputs are thought to make more numerous excitatory synapses, located more proximally on the dendrites of ChIs (Lapper and Bolam, 1992; Dimova et al., 1993; Thomas et al., 2000). The centromedian/parafascicular (CM/Pf) region of thalamus in the primates, homologous to the lateral and medial Pf thalamic nuclei in the rat, constitutes a major excitatory glutamatergic input to the striatum (Smith et al., 2004, 2009). Inactivation of CM-Pf by local infusion of muscimol has been shown to attenuate the pause and rebound, but not the initial excitation responses, of TANs to sensory cues (Matsumoto et al., 2001), indicating thalamic input is necessary for the expression of the full three-phase pause response. Removing thalamic input can also reduce firing rate and intrinsic activity of ChIs in rats (Bradfield et al., 2013). Electrical stimulation of thalamus *in vivo* can induce pauses in ChIs in rats (Doig et al., 2014). This is backed up by *ex vivo* slice studies which demonstrate that stimulation of thalamic axons electrically or Pf inputs using optogenetics can induce a pause in the ChIs (Ding et al., 2010; Kosillo et al., 2016; Figure 2D). Visual stimulation *in vivo*, which co-activates the thalamostriatal and nigrostriatal pathways can also induce a pause response in ChIs in rats (Schulz et al., 2011).

However, no unequivocal cellular mechanism emerges for thalamus-induced pauses in ChIs. The pause phase has been proposed to be dependent on initial excitation by thalamus from a study in anesthetized rats (Schulz et al., 2011), but yet a monkey study has suggested oppositely that it is the pause and rebound that are removed following thalamus inactivation, independently from initial excitation phases (Matsumoto et al., 2001). Excitatory input has also been proposed to be able to drive a ChI pause in slices in a dopamine-dependent manner through which synchronized activation of ChIs can drive DA release (Cachope et al., 2012; Threlfell et al., 2012) and in turn promote inhibition of ChIs (Ding et al., 2010). But yet, not all pause responses show initial excitation as discussed above. So, if thalamic inputs are required for pause responses *in vivo*, it would seem to be through a mechanism that does not evoke initial spiking, and may not then depend on activation of DA release.

### Unknown GABA Input

ChIs can inhibit their neighboring ChIs via a GABA-dependent mechanism and a currently unidentified striatal source (Sullivan et al., 2008). When an electrical stimulation is applied to the striatum *ex vivo*, ChIs receive an acetylcholine-dependent GABAergic inhibitory current with a latency of ~7–11 ms (Sullivan et al., 2008). This inhibitory current could, in theory, induce a pause in ChIs in response to an initial excitation of a subpopulation of ChIs. However, the presumed GABAergic neuron that underlies this mechanism have not yet been unidentified. Moreover, a pause generated by this input would depend on an initial excitation, and can therefore not explain fully why pauses can occur without apparent initial excitation, or why pause length is variant when appetitive or aversive stimuli are given (Ravel et al., 2003).

All the mechanisms outlined above have been considered for their potential contribution to the stereotyped triphasic pause response in ChIs. We add further that a second pause following the rebound has repeatedly been observed in behavioral tasks (Ravel et al., 1999, 2003; Apicella et al., 2011), and note that none of these varied hypothesis can readily explain this second pause.

## Summary and Perspective

In summary, a range of afferent inputs to striatum can induce some form or component of the pause response in ChIs *in vivo* and/or *ex vivo*, particularly midbrain dopamine and GABA inputs, and corticostriatal and thalamostriatal inputs. But yet no single source appears to be able to account for all features of pauses, temporally or spatially. It is possible therefore that the pause response is driven by a multifactorial influence of all of these inputs, which may then be required to act with specific sequence and timing to generate different components of the pause.

Alternatively, the possibility cannot be excluded that there might be an underpinning mechanism which provides a unifying explanation for all pause responses observed. In that case, the potential mechanism needs to fit all the common characteristics of the pause response, i.e., afford a synchronization of all three phases of the pause responses, to be recruited across the striatum, in both hemispheres, and with the pause developing in amplitude but not necessarily duration during learning. In addition, this potential unifying underlying mechanism should to be able to explain the variations in pauses responses i.e., varying length of pause response in aversive vs. appetitive tasks, and a response of TANs to some but not other stimuli. The inhibition of TAN activity caused by this mechanism should operate even when an enhanced initial excitation is missing, and yet it should be responsive to changes in excitation as well as to neuromodulatory inputs. The inhibition of TAN activity generated should also be able to outlive the cycle of one interspike interval, and even contribute to a second pause after a rebound.

Despite being studied for more than 20 years, the pause response of TANs is still incompletely understood. The pause response in TANs is proposed to play critical roles in shaping striatal output, dopamine signals and learning. An improved understanding of the mechanisms underlying pause responses will help us to better comprehend the powerful functions of ChIs in the striatum.

## Author Contributions

YFZ and SJC co-wrote the manuscript.

## Conflict of Interest Statement

The authors declare that the research was conducted in the absence of any commercial or financial relationships that could be construed as a potential conflict of interest.
